# The American Medical Association at New Orleans

**Published:** 1903-04

**Authors:** 


### The American Medical Association at New Orleans.

THREE times since its establishment the national medical body has appointed its meetings at New Orleans, once in 1869, again in 1885, and now for May 5-8, 1903. It was not our privilege to attend the first time, but we had the good fortune to be present on the second occasion, hence, we can predict with the knowledge gained by experience that the approaching meeting will be one of the most enjoyable in the history of the association.

New Orleans, famous for its hospitalities, is at its best in May, when its verdure is fresh, when the fragrance of magnolias fills every air, and the hearts of its citizens, in the effulgence of the springtime, beat a responsive welcome to every coming guest. The city has grown since the last visit of the association and, great as were its attentions then, its reception to the profession of medicine in May will be proffered on a much wider scale.

The physicians of New Orleans are accustomed to extend generous greetings to their guests, and under the chairmanship of Dr. Isadore Dyer, an experienced and accomplished executive, something entirely beyond the ordinary may be expected, for, indeed, as given out, the plans indicate a wider-embracing scope than ever laid out on any previous occasion. The attendance bids fair to be the largest ever registered at any gathering of medical men in this country.

Once having settled in the affirmative the question of going, it becomes next of great importance to arrange details of the journey and the place of living in the Crescent City. For those residing in or near Buffalo the Illinois Central Railroad offers the most direct, attractive, and economic route of travel. The first stage of the journey will be either via Cincinnati or Chicago, the next to Memphis, and, finally, to New Orleans, the time occupied being about 48 hours. The fare for the round trip is $29, or $30.50, according to the route traveled from Buffalo to Cincinnati. This, however, can be arranged best by a visit to the office of Mr. G. B. Wyllie, at No. 210 Ellicott Square, the local representative of the road, who will gladly furnish any information whether tickets are purchased or not.

Hotel or other accommodations may be secured by addressing Dr. Isadore Dyer, chairman of the committee of arrangements, 124 Baronne Street, New Orleans, who will turn such requests over to the proper subcommittee for attention and answer. It is advisable that such applications be filed early in order to obtain desirable quarters.

